# Mapping fertility rates at national, sub-national, and local levels in Ethiopia between 2000 and 2019

**DOI:** 10.3389/fpubh.2024.1363284

**Published:** 2024-09-23

**Authors:** Biruk Beletew Abate, Ashenafi Kibret Sendekie, Mulat Ayele, Eyob Shitie Lake, Tilahun Wodaynew, Befkad Derese Tilahun, Molla Azmeraw, Tesfaye Engdaw Habtie, Muluemebet Kassa, Melesse Abiye Munie, Dessie Temesgen, Abebe Merchaw, Addis Wondimagegn Alamaw, Alemu Birara Zemariam, Tegene Atamenta Kitaw, Amare Kassaw, Ayelign Mengesha Kassie, Gizachew Yilak, Mulat Awoke Kassa, Gebremeskel Abebe Kibret, Muluken Chanie Agimas, Fasikaw Kebede Bizuneh, Molalign Aligaz Adisu, Bogale Molla Woreta, Kefyalew Addis Alene

**Affiliations:** ^1^School of Population Health, Curtin University, Bentley, WA, Australia; ^2^College of Medicine and Health Sciences, Woldia University, Woldia, Ethiopia; ^3^Curtin Medical School, Faculty of Health Sciences, Curtin University, Bentley, WA, Australia; ^4^Department of Clinical Pharmacy, College of Medicine and Health Sciences, University of Gondar, Gondar, Ethiopia; ^5^Department of Nursing, College of Health Science, Debre Tabor University, Gondar, Ethiopia; ^6^School of Public Health, The University of Queensland, Brisbane, QLD, Australia; ^7^Department of Epidemiology and Biostatistics, Institute of Public Health, College of Medicine and Health Sciences, University of Gondar, Gondar, Ethiopia; ^8^Faculty of Health Sciences, Curtin University, Bentley, WA, Australia; ^9^Geospatial and Tuberculosis Research Team, Telethon Kids Institute, Nedlands, WA, Australia

**Keywords:** mapping, fertility rate, spatial analysis, trends, Ethiopia

## Abstract

**Background:**

Fertility rates are key indicators of population health and demographic change, influencing economic development, healthcare planning, and social policies. Understanding subnational variation in fertility rate is important for effective geographical targeting and policy prioritization. This study aimed to identify geographic variation, trends, and determinants of fertility rates in Ethiopia over the past two decades.

**Methods:**

We estimated total fertility rates (TFRs) and age-specific fertility rates (ASFRs) using five nationally representative cross-sectional Demographic and Health Surveys collected in Ethiopia between 2000 and 2019. ASFRs represent the number of live births per 1,000 women aged 15 to 49 during the 3 years before each survey, while TFRs indicate the average number of children a woman would have by the end of her reproductive years, calculated as the sum of ASFRs over five-year intervals. We developed model-based geostatistics by incorporating demographic and healthcare access data with spatial random fields to produce high-resolution fertility rate maps. These results were then aggregated to produce fertility rate estimates at local, sub-national, and national levels in Ethiopia.

**Results:**

The national TFR gradually declined from 4.8 live births in 2000 to 4.2 live births in 2019, but it is still above the replacement level of 2.1 children per woman. There were sub-national and local variations in TFR, ranging from 5.7 live births in Somalia and 5.3 Oromia regions to 2.7 live births in Addis Ababa and 3.6 live births Dire Dawa cities. Geographical areas with high TFR were mostly associated with a high proportion of Muslim women and low access to health facilities.

**Conclusion:**

Despite a decline in fertility rates among women of reproductive age over the past two decades, marked spatial variation persists at sub-national and local levels in Ethiopia, with demographic factors determining the spatial distribution and rate of decline, highlighting the need for tailored programs and strategies in high-fertility areas to increase access to family planning.

## Introduction

Fertility rates are critical indicators of population health and demographic change, influencing economic development, healthcare planning, and social policies. They provide essential information about the population’s capacity to reproduce and its growth potential, shaping the age structure and demographic dynamics of a country (1). Understanding fertility rates is crucial not only for predicting future population trends but also for informing policy decisions that aim to enhance economic development, optimize healthcare services, and address social needs (2).

Globally, total fertility rates (TFRs) have declined over the past century, but the pace and timing of this decline have varied significantly across countries. High-income countries have generally experienced more rapid declines in fertility rates, while low-income countries have seen more stagnant or even increasing rates (3). Despite the overall decline in TFRs, the world’s population continues to grow, with a global population reaching 8.0 billion in 2023 and projected to hit 9.7 billion by 2050 (2). Developing countries tend to have higher fertility rates compared to developed countries (4). For example, Sub-Saharan Africa has an average of 5.1 births per woman, nearly double the rates of South Asia (2.8) and Latin America and the Caribbean (2.2) (5, 6).

Ethiopia has experienced considerable changes in its fertility rates over the past two decades. National data indicate a steady increase in fertility rates, yet this overall trend masks significant subnational variations. After an increase in fertility during the 1960s and 70s, a gradual decline began in the 1980s and 90s (7), resulting in a significant decrease in TFR from 5.5 children per woman in 2000 to 4.6 in 2016 (1). However, substantial regional disparities persist within Ethiopia. In 2016, Addis Ababa had the lowest TFR at 1.8 children per woman, while Somali region had the highest rate at 7.2 children per woman (1). Geographical variations in fertility rates are influenced by a complex interplay of demographic, socioeconomic, and cultural factors. Understanding the geographical distribution and the determinants of fertility rates can help policymakers develop targeted interventions to address specific needs, promote balanced population growth, and improve reproductive health outcomes. This study aimed to investigate the spatial distribution, trends, and determinants of fertility rates in Ethiopia over the past two decades using advanced spatial analysis techniques. The findings provided high-resolution maps of fertility rates that can guide policy decisions and resource allocation, contributing to more effective and equitable health and development strategies in Ethiopia.

## Methods

### Study settings

The study was conducted in Ethiopia, the second-most populous country in Africa with an estimated population size of more than 120 million people in 2022 and a surface area of approximately 1.1 million square kilometers. There are marked differences in population density, socioeconomic conditions, and geographical features across the country. More than 84% of the population resides in rural parts of the country with low healthcare access including contraceptive services. The country has three administrative levels including region, zone, and districts.

### Data source

Data for total fertility rate (TFR) and age-specific fertility rate (ASFR) were obtained from the Ethiopian Demographic and Health Survey (EDHS), which consists of five nationally representative cross-sectional surveys conducted in 2000, 2005, 2011, 2016, and 2019. ASFR represents the number of live births to women aged 15 to 49 during the 3 years before each survey. The TFR indicates the average number of children a woman would have by the end of her reproductive years, calculated as the sum of ASFRs over five-year intervals. ASFR and TFR were used because they provide a comprehensive understanding of fertility patterns across different age groups and the overall reproductive behavior of women, making them more informative and accurate compared to single-point estimates of fertility.

The EDHS used a two-stage cluster sampling design with stratification into urban and rural areas. In the initial phase of the selection process, primary sampling units, often census areas, were chosen based on their size, ensuring that larger areas had a higher chance of being selected within each group. Next, a list of households was created within these selected areas, and a set number of households were picked using a systematic method where each had an equal chance of being chosen. Because of this selection method, sampling weights were applied during the analysis to account for any differences in sampling rates among various groups, ensuring that the results were accurate and representative. After data cleaning and applying sampling weights, the final analytic sample included 37,251 women of reproductive age (15–49 years). The EDHS contains geographic information with latitude and longitude coordinates for each surveyed clusters (enumeration areas), enabling spatial analysis and mapping of fertility rate at subnational levels. In total, we gathered data from 2,618 unique locations for women aged 15–49 years between 2000 and 2019.

### Covariate data

In addition to demographic data gathered through the EDHS, we also compiled environmental covariate data from publicly available sources that we extracted to EDHS cluster locations. Because these locations are displaced, we averaged geospatial covariates to 2 km and 5 km buffers for urban and rural locations, respectively. Covariates were limited to those for which country-wide representative data were available at a high resolution and that are plausibly associated with fertility rate. Data on travel time to the nearest city were obtained from Malaria Atlas Project (MAP) (8). Altitude data were obtained from the Shuttle Radar Topography Mission (SRTM) (9). Population density, estimated as the number of people per grid, was obtained from WorldPop (10). Distance to the nearest health facilities was also obtained from MAP. Data on religion were obtained from the EDHS, and to estimate the proportion of Muslim women at a high resolution, we employed Bayesian Kriging interpolation techniques (11, 12). To do this we first defined the Kriging model, applied Bayesian inference for parameter estimation, integrated the covariate, and used Bayesian methods to update the model with the data, allowing us to explore the impact of the proportion of Muslims on the fertility rates. This advanced statistical method allowed us to create detailed maps showing the distribution of Muslim women across various regions, providing a more accurate depiction of religious demographics to include in our spatial model. The covariate maps used in our models are provided in the supplementary information. Before fitting multivariable models, we checked for multi-collinearity between covariates using a variance correlation matrix.

### Geoprocessing

As the raster of the covariates were at different resolutions, we standardized them to a common resolution of approximately 1 × 1 km. We achieved this by performing resampling and aggregation as pre-processing steps before combining the data, following previously described methods (13–15). All variables were resampled and clipped to the extent of Ethiopia. This approach allowed us to align all the covariate data at the desired resolution.

### Geospatial analysis

Bayesian geostatistical models were fitted to analyze Ethiopia’s total fertility rate and age-specific fertility rate, utilizing integrated nested Laplace approximation (INLA) for model inference and prediction. INLA applies the Stochastic Partial Differential Equation (SPDE) method to account for spatial interaction effects, estimating a continuous Gaussian Markov Random Field (GMRF) with spatial correlations between locations defined by the Matérn correlation function. The Matérn correlation function is a popular spatial model in geostatistics and Kriging. It determines how the correlation between points declines with distance, using parameters for smoothness and range, and can accommodate various levels of spatial smoothness (16). The Bayesian models were created by estimating a Gaussian generalized additive model (GAM) with and without spatially correlated random effects. The Gaussian GAM is defined as follows:

$$y_{i}=\beta_{0}+\overset{m}{\underset{j=1}{\sum }}\beta_{j}X_{j,i}+\overset{l}{\underset{k=1}{\sum }}f_{k}\left(X_{k,i}\right)+\mu_{i}+\in_{i}$$

where $y_{i}$ denotes the observed TFR or ASFR at the location i , $\beta_{0}$ is the intercept, $X_{j,i}$ and $X_{k,i}$ are the ith and kth covariates at location i , $\beta_{j}$ are the beta coefficients, $f_{k}$ are the smooth functions for the kth covariates, $\mu_{i}$ is the spatial random effect at the location i , and $\in_{i}$ is the residual error. The spatial random effect $\mu_{i}$ was calculated using the SPDE approach. This method involves representing the continuous spatial process as a GMRF by discretizing the study areas into a mesh of triangular elements. The Matern correlation function, which defines the spatial correlation structure, was used to model the covariance between points in the spatial field. The parameters of the Matern function, such as the range and smoothness, were estimated as part of the INLA process, allowing for the incorporation of spatial dependence into the model. Because no prior information was available, a Gaussian prior distribution with a mean of zero (default no effect prior unless data is informative) was applied for all the model parameters. The posterior mean, standard deviation, and 95% credible intervals were estimated for all the parameters. The posterior distributions generated from the model, showing the predicted TFR and ASFR, were mapped using ArcGIS Pro (ESRI, Redlands, CA). These maps provide a detailed spatial understanding of fertility rates across Ethiopia, allowing for more targeted and effective interventions. We aggregated the pixel-level estimates to produce fertility rate estimates at local, sub-national, and national levels in Ethiopia. This process summarized detailed pixel-level data into larger geographic units, ensuring our findings are relevant and useful for policymakers and stakeholders at various administrative levels. We have checked the multicollinearity between covariates. The correlation coefficient for all covariates is less than 0.7 and is considered as low suggesting the absence of multicollinearity (Figure S1).

## Results

**Figure 1:**
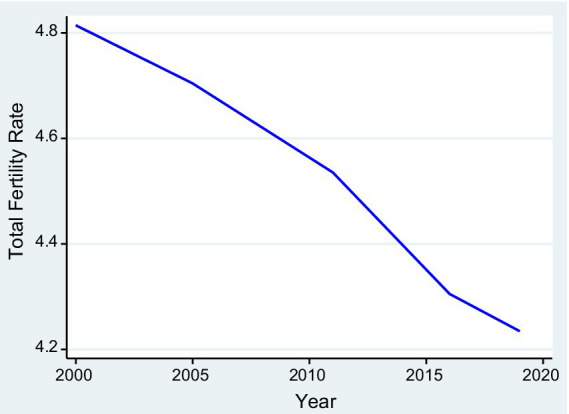
Average national-level trends of total fertility rates in Ethiopia.

**Figure 2:**
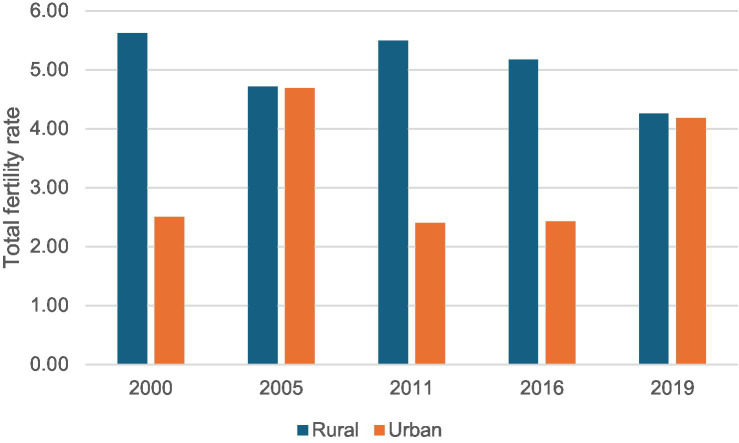
Total fertility rate by rural and urban across all surveys.

We analyzed 2,618 nationally representative survey clusters from the EDHS for the period 2010–2019. Figure 1 describes the national-level trends of the TFR in Ethiopia from 2000 to 2019. The TFR was 4.8 live births per woman in 2000, and over two decades, it decreased by 12.5% to 4.2 live births per woman by 2019, showing a gradual decline while remaining above the replacement level of 2.1 children per woman. The ASFR was 0.96 in 2000 and decreased to 0.84 in 2019. The temporal trends in the decline of live births per woman mask significant heterogeneity at the subnational level. Figure 2 shows that TFR in Ethiopia from 2000 to 2019 consistently higher in rural areas compared to urban areas.

### Spatial distribution of fertility rate in Ethiopia

**Figure 3:**
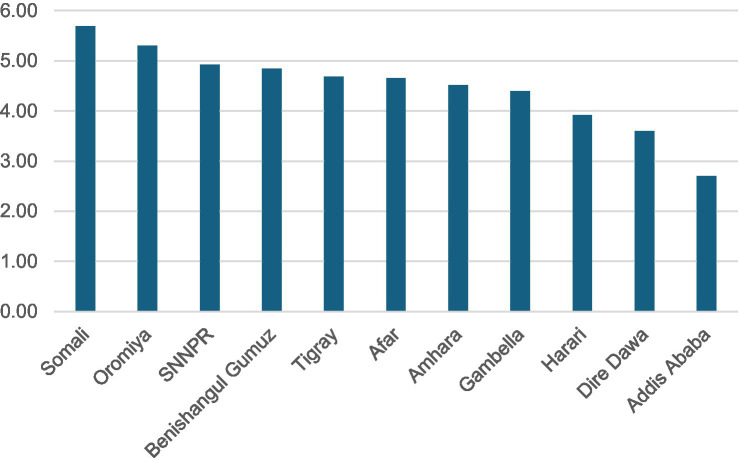
Total fertility rates by regions in Ethiopia.

**Figure 4:**
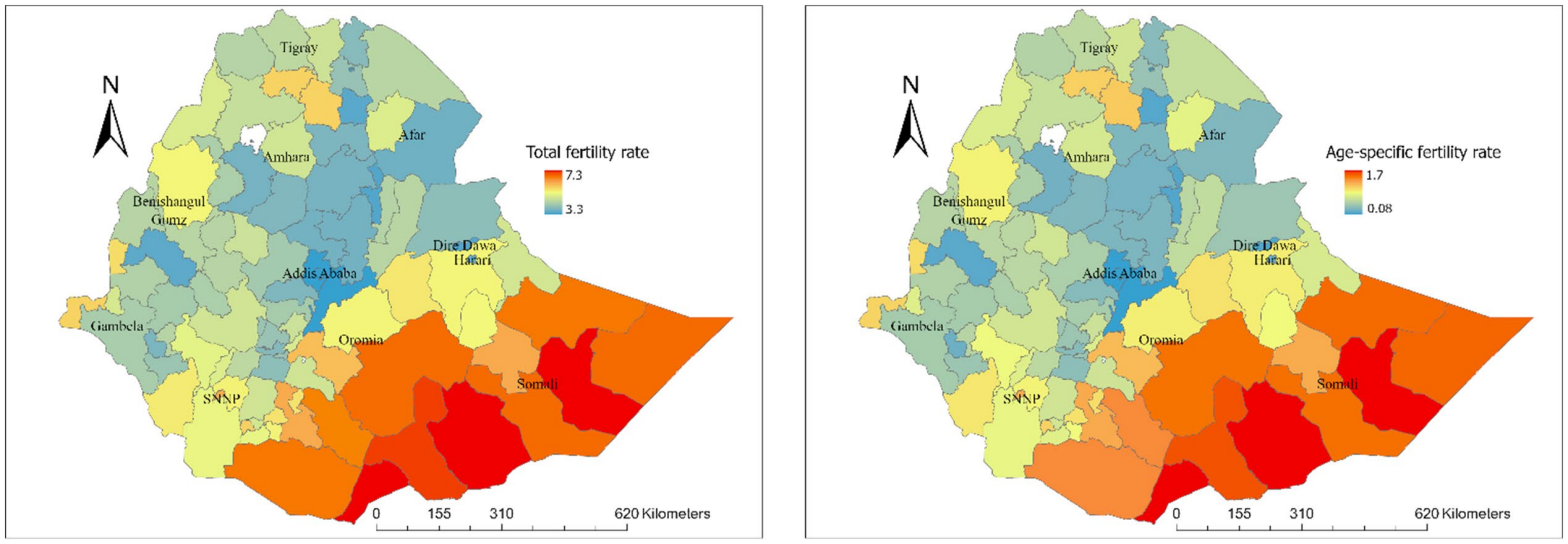
Total and age-specific fertility rate by zone level in Ethiopia.

**Figure 5:**
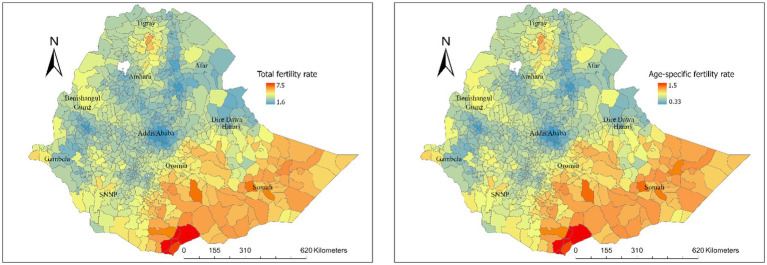
Total and age-specific fertility rate by district level in Ethiopia.

**Figure 6:**
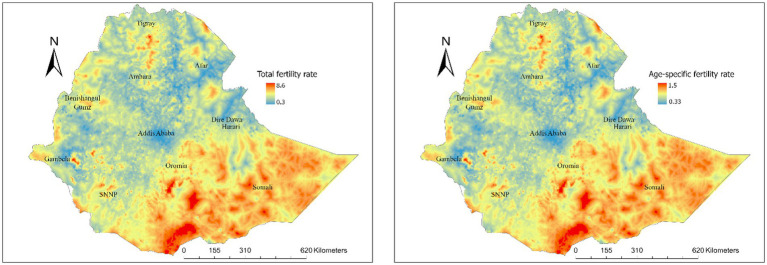
Total and age-specific fertility rate at pixel level in Ethiopia.

Substantial spatial variations in TFR were observed at regional, zonal, district and pixel levels in Ethiopia. Regional variations in TFR ranged from 5.7 live births in the Somali region and 5.3 in Oromia to 2.7 in Addis Ababa and 3.6 in Dire Dawa (Figure 3). At the zone level, total fertility rates ranged from 3.3 to 7.3 live births, and age-specific fertility rates ranged from 0.08 to 1.7 live births (Figure 4). At the district level, total fertility rates varied from 1.6 to 7.5 live births, with age-specific rates between 0.33 and 1.5 live births (Figure 5). At the pixel level, total fertility rates ranged from 0.3 to 8.6 live births, and age-specific rates also ranged from 0.33 to 1.5 live births (Figure 6).

### Factors associated with total and age-specific fertility rate

Table 1 shows the factors associated with the TFR and ASFR derived from the Bayesian geostatistical model. Distance to the city was positively associated with both total fertility rate (*β*: 0.42; 95% CrI: 0.23, 0.62) and age-specific fertility rate (*β*: 0.08: 95% CrI: 0.04, 0.12). The proportion of Muslim women was also positively associated with both the total fertility rate (*β*: 0.37: 95% CrI: 0.32, 0.42) and age-specific fertility rates (*β*: 0.07; 95% CrI: 0.06, 0.08). In contrast, the population density was negatively associated with both total fertility rate (*β*: −0.01; 95% CrI: −0.02, −0.01), and age-specific fertility rates (*β*: −0.003; 95% CrI: −0.004, −0.002) indicating lower fertility rates in more densely populated areas (Figure S2).

**Table 1:** Factors associated with total fertility rate and age-specific fertility rate.

Variables	Total fertility rate	Age-specific fertility rate
Distance to health facility	0.17 (−0.11, 0.45)	0.03 (−0.02, 0.09)
Distance to city	**0.42 (0.23, 0.62)**	**0.08 (0.04, 0.12)**
Proportion of Muslim women	**0.37 (0.32, 0.42)**	**0.07 (0.06, 0.08)**
Altitude	0.14 (−0.02, 0.30)	0.03 (0.003, 0.06)
Population density	−**0.01 (−0.02, −0.01)**	−**0.003 (−0.004, −0.002)**

Bold values indicates statistically significant association with P-value less than 0.05.

## Discussion

This study applied an advanced geospatial analysis approach to map fertility rates at national, sub-national, and local levels in Ethiopia between 2000 and 2019. The fertility rates in Ethiopia have shown a consistent decline over the last two decades, though they remain above the replacement level. The declining trend might be attributed to urbanization, increased focus on women’s education, and improved healthcare access, which typically result in delayed marriages and higher contraceptive use. Contraceptive coverage in Ethiopia increased from 8.1% in 2000 to 41.4% in 2019. This declined trend is consistent with findings from other studies conducted in Uganda (17) and Europe (18). Evidence also showed a low fertility in some Asian countries such as Singapore, Japan, and China, where government policies aimed at population control, like ‘one-child policy’, have been implemented (19).

Despite a gradual decline in fertility rates at the national level, substantial regional disparities have been observed, with rates ranging from 5.7 live births per woman in the Somalia region and 5.3 in Oromia to 3.6 Dire Dawa city and 2.7 in Addis Ababa. This regional discrepancy might be due to unequal access and utilization of family planning services, with high contraceptive use in major cities like Addis Ababa (20). Studies show a strong correlation between contraceptive use and fertility decline (21). The increased contraceptive use in Addis Ababa city and other regions such as Amhara region may be part of government efforts to manage population growth through targeted strategies (22). Ensuring equitable access to family planning services and addressing regional socioeconomic disparities is crucial for achieving a consistent decline in fertility rates nationwide.

Out study also showed that women in rural areas are more likely to have higher fertility rates compared to their urban counterparts (23). Previous studies conducted in Sub-Saharan Africa (SSA) also indicate significant disparities in fertility rates between urban and rural areas (24, 25). This disparity may be due to easier access to contraception and higher educational attainment in urban areas (26–30). Additionally, women in rural areas tend to marry earlier, with an average marriage age of 16.1 years compared to 19.4 years in urban areas (31). These findings suggest that improving access to family planning services and education in rural areas could help reduce fertility rates and address regional disparities.

Furthermore, the fertility rate also varied by religion, with areas having a high proportion of Muslim women exhibiting higher fertility rates. This variation may be influenced by cultural norms and practices regarding family size and contraceptive use within different religious groups (32, 33). These findings showed the importance of considering religious and cultural contexts when designing and implementing family planning programs. Tailored interventions that respect and address specific cultural and religious beliefs are crucial for effectively managing fertility rates and promoting reproductive health across diverse communities.

Another important finding of our study is the positive association between distance to the city and fertility rates. Women living further from urban centers tend to have higher fertility rates, likely due to limited access to healthcare facilities, family planning services, and educational opportunities, which are more readily available in cities. This relationship is further explained by the increased costs of transportation as travel time to the nearest cities increases. In low-income African countries, research has consistently shown a significant link between geographic accessibility to healthcare facilities and the utilization of maternal and child health services (34). Urban areas often provide better economic opportunities and social services, leading to delayed marriages and smaller family sizes. This finding shows the necessity for improved infrastructure and service delivery in rural areas to bridge the gap between urban and rural fertility rates. Enhancing accessibility to healthcare and education in remote regions is essential for addressing these disparities and promoting equitable reproductive health outcomes across the country (35–37). This study has significant implications for policymakers and health program designers, emphasizing the need for geographically targeted and integrated fertility control programs that address ecological and religious disparities.

Our study has some limitations. One limitation is the reliance on cross-sectional data from the EDHS, which may not fully capture the temporal dynamics of fertility trends and their determinants. The relatively smaller sample size in the EDHS 2019 dataset may have slightly affected the comparability of findings. Additionally, the study also does not account for potential confounding factors such as cultural practices, economic variations, and local policy differences that might influence fertility rates independently of geographic factors. Furthermore, Ethiopia’s challenging topography and infrastructure limitations might affect the accuracy of spatial data, leading to potential misclassification of accessibility levels.

## Conclusion

The fertility rate among women of reproductive age in Ethiopia gradually declined from 2000 to 2019, yet significant spatial disparities persist at regional and local levels, influenced by demographic factors. Tailored programs and strategies are essential in high-fertility areas to improve access to family planning and address unequal fertility trends across different regions, religions, residential settings. Further research is needed to identify independent factors influencing fertility rates and to understand regional and religious disparities, incorporating social, economic, and other characteristics at both national and subnational levels.

## Data Availability

The original contributions presented in the study are included in the article/Supplementary material, further inquiries can be directed to the corresponding author.
