# Supplementary material for: Mapping fertility rates at national, sub-national, and local levels in Ethiopia between 2000 and 2019
Source: Front Public Health. 2024 Sep 23;12:1363284. doi: 10.3389/fpubh.2024.1363284 (PMC11459572; doi:10.3389/fpubh.2024.1363284)
Supplement: Supplementary file 1 [file Image_1.pdf]

Supplementary Figures

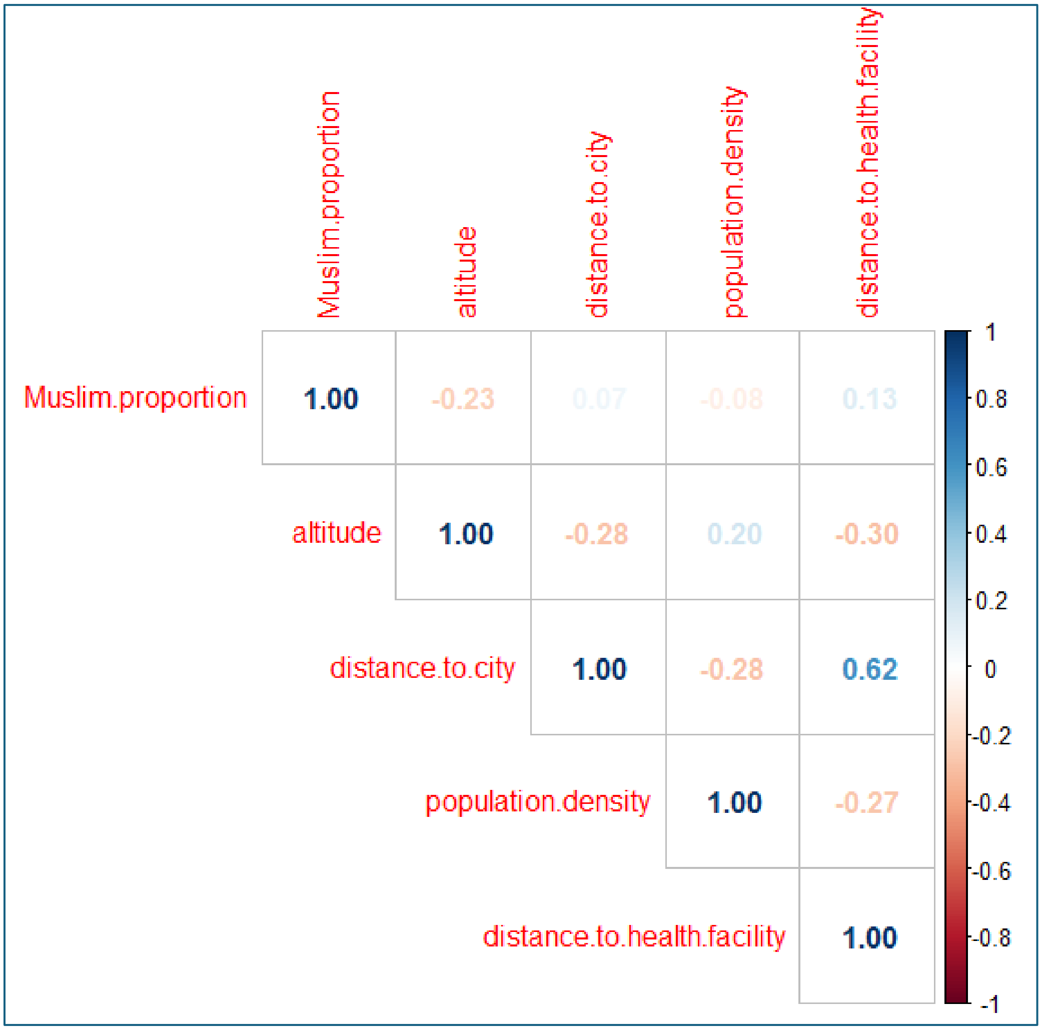

Figure S1: Correlation matrix to check multicollinearity between covariates.

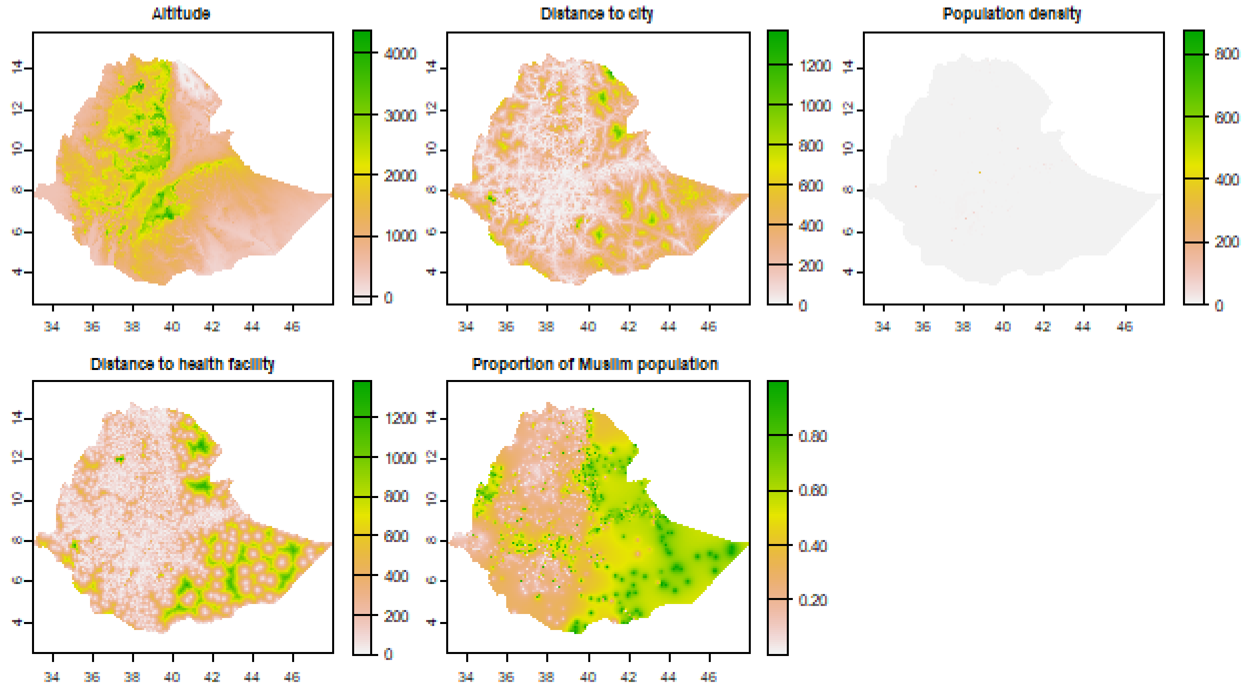

Figure S2: Covariates included in the geospatial modelling of total fertility rate in Ethiopia
